# Interrogation of Interfering Factors in ELISA Detecting Angiotensin Receptor Antibodies and Specificity Validation Using the Adsorption Elution Crossmatch (AXE) Technique

**DOI:** 10.1111/tan.70268

**Published:** 2025-06-08

**Authors:** Adak Karamafrooz, Julie Nguyen, Hong Ma, Dave Lowe, Michael Trinh, Rui Pei, Robert Carroll

**Affiliations:** ^1^ R&D Biochemistry Department One Lambda Inc. West Hills California USA; ^2^ Department of Health Sciences University of South Australia Adelaide South Australia USA; ^3^ Transplantation Immunogenetics Service Australian Red Cross Lifeblood Adelaide Australia

## Abstract

Accurate detection of HLAs and non‐HLA antigens is critical for managing long‐term allograft transplantation, particularly in the context of hyperacute, acute, and chronic allograft rejection. Recent studies have identified the role of non‐HLA antibodies, such as those against Angiotensin II Type 1 receptor (AT_1_R) in transplant rejection. The enzyme‐linked immunosorbent assay (ELISA) is the primary method for measuring AT_1_R‐specific antibodies (AT_1_R‐Ab), offering high specificity and reasonable sensitivity. Despite its widespread use in clinical settings, some reports have suggested that pre‐treating the samples with latex beads can eliminate the detection signal in the CellTrend AT_1_R ELISA assay, potentially raising concerns over false reactivity in the assay. In this study, we demonstrate that the bovine serum albumin (BSA) present in the adsorb out beads (AOB) buffer, even at a dilution of 10^−6^, plays a key role in signal elimination in the CellTrend AT_1_R‐Ab detection kit. Additionally, we evaluated the performance of the CellTrend kit and an in‐house affinity‐purified AT_1_R ELISA in detecting eluted AT_1_R‐Abs from live cells using the adsorption crossmatch and elution (AXE) technique, which achieved a median elution efficiency of 30% when tested on the CellTrend ELISA platform. Our findings support that the CellTrend ELISA kit accurately detects anti‐AT_1_R antibodies that bind to the active form of AT_1_R. However, serum treatments containing BSA interfere with the antibody–antigen capture interface, leading to signal suppression.

## 1 Introduction

Rapid and accurate methods for the detection of HLAs and non‐HLA antigens associated with the pathogenesis of hyperacute, acute, and chronic allograft rejection are clinically important for the successful management of long‐term allograft transplantations [1, 2, 3]. HLA antibodies are major contributors to the pathophysiology of antibody‐mediated rejection (ABMR) and play a role in the longevity of transplantation outcomes. Several clinical cases of allograft rejection associated with the absence of anti‐HLA antibodies have recently been identified and recognised [4]. While some non‐HLA antigens have been implicated in transplant pathogenesis [5], few diagnostic screening tools have been developed to effectively detect and monitor these antibodies. Among non‐HLA antigens, antibodies against Angiotensin II Type 1 receptor (AT_1_R) have been extensively investigated and shown to be associated with vascular inflammation, which leads to refractory vascular rejection and eventual allograft loss, enhancing the mortality risk in kidney transplant patients [6, 7, 8, 9, 10]. Whether the accumulation of antibodies against AT_1_R (AT_1_R‐Ab) acts against (allo‐) or (auto‐) antigens, its relative concentration plays an important role in the success of transplant outcome [9, 11].

The Human AT_1_R ELISA is the most widely utilised assay for quantifying AT_1_R‐Ab levels, demonstrating 100% specificity and 88% sensitivity in various clinical studies on kidney transplantation [8, 12, 13, 14, 15]. The AT_1_R ELISA preparation involves coating the 96‐well plate with whole cell lysate extract derived from CHO cells transfected with human AT_1_R [12, 14]. Patient serum is then applied, allowing AT_1_R‐specific antibodies to bind to the coated plate. Detection is achieved using a secondary antibody conjugated to horseradish peroxidase (HRP), which catalyses the conversion of the chromogenic substrate TMB (3,3′,5,5′‐tetramethylbenzidine) into a blue‐coloured product. The resulting signal is quantified calorimetrically using an ELISA plate reader, typically at a wavelength of 450 nm [16]. The measured absorbance is then compared against a standard curve ranging from 0 (0 U) to 40 unit/mL (40 U) to obtain the index value of AT_1_R‐Ab semi‐quantitatively (CellTrend, EIA for Quantitation Determination of Anti‐Angiotensin II Receptor 1 [AT‐1]‐Antibodies) [17].

Currently, no standardised cutoff value has been established for defining AT_1_R‐Ab positivity. Clinical studies have reported varying pathological thresholds, ranging from 9 to 30 U/mL [12, 13, 14, 15, 18]. Several factors may contribute to these discrepancies, including the limited availability of non‐enriched AT_1_R derived from whole‐cell lysates, in contrast to affinity‐purified AT_1_R antigens immobilised on ELISA plates. Additionally, the inclusion of specific reagents such as bovine serum albumin (BSA) or absorb out beads (AOBs) as blocking agents to mitigate background noise affects the binding avidity between AT_1_R‐Ab and its target AT_1_R antigen during the assay [19, 20].

Improving the accuracy and sensitivity of AT_1_R‐Ab detection in transplant patient sera is critical for enhancing clinical consistency and assay reproducibility. In this study, we assessed the sensitivity of a commercial AT_1_R‐Ab detection kit (CellTrend) using AT_1_R‐specific antibodies eluted from live cells via the adsorption crossmatch and elution (AXE) technique [21]. Furthermore, we evaluated the specificity and sensitivity of an affinity‐purified, constitutively active AT_1_R [22, 23, 24] on the ELISA platform and compared its antibody‐binding affinity to that of the wild‐type AT_1_R.

## 2 Materials and Methods

### 2.1 Transfection and Expression of AT_1_R

T‐rex 293 adherent cells were maintained in DMEM high glucose supplemented with 10% FBS, 1% penicillin–streptomycin, and GlutaMAX at 37°C and 5% CO_2_ in a humidified incubator. T‐Rex 293 cells were transfected with AT_1_R wild type (AT_1_R‐WT) or AT_1_R active form using a Neon machine electroporation system. Stably integrated pcDNA3.1‐Zeo‐tetO‐AT_1_R constructs were confirmed for plasmid integration using flow cytometry and maintained with 400 μg/mL zeocin as the selection drug. Cells were induced using 1 μg doxycycline in the presence of 5 mM sodium butyrate and assayed for AT_1_R expression on the cell surface using AT_1_R‐specific in‐house or commercial antibodies at least 24 h post‐induction.

### 2.2 AT_1_R Surface Expression

Induced (using doxycycline) and uninduced Trex‐293 cells were harvested after 24 or 48 h, and cell pellets to 2 × 10^7^ cells/mL were resuspended in staining buffer containing the PBS and primary conjugated anti AT_1_R‐Ab (488‐ Alexa Fluor 488 conjugated 2C4) or primary anti AT_1_R‐Ab (SC G‐3) for 30 min at 4°C, followed by incubation with the appropriate secondary conjugated antibody. The cells were then washed and resuspended in PBS, filtered for cell clumps, and analysed with FACS Lyrics flow cytometry. For sorting Trex‐293 cells expressing AT_1_R, antibody incubation steps were performed as described above with an additional last step of incubating cells in Gibco cell dissociation buffer.

### 2.3 Purification of AT_1_R Using Affinity Chromatography

Trex‐293 transfected cells were maintained in DMEM high glucose supplemented with 10% FBS, 1% penicillin–streptomycin, and GlutaMAX at 37°C and 5% CO_2_ until they reached about 80% confluency. Cells were harvested 48 h post‐induction and tested for AT_1_R expression using surface staining followed by flow cytometry. For AT_1_R purification, we resuspended the pellet in the hypotonic buffer following the protocol by Wingler et al. [25], with minor modifications. The purification was performed using affinity chromatography for tagged and untagged AT_1_R constructs, and all steps were performed at 4°C. AT_1_R was eluted using three bed volumes of 0.1 M glycine pH 2.0–2.8 and immediately neutralised with 1 M Tris, pH 9.5. The eluted fraction was buffer exchanged with buffer (20 mM HEPES, pH 7.5, 500 mM NaCl, 0.01% MNG, 0.004% [w/v] CHS, protease inhibitor) and analysed using BCA assay, western blot, and LC–MS mass spectrometry protein sequencing (Supporting Information).

### 2.4 In‐House ELISA Tray for Detection of Anti‐AT_1_R Autoantibodies

(a) Stably transfected cells with AT_1_R inducible construct were seeded at a density of 0.05 × 10^6^ per well on poly‐d‐lysine‐coated 96 well plates, induced 24 h later using 1 μg doxycycline and fixed with 4% formaldehyde. Following three washes with PBS, the coated cells were used to capture antibodies from a panel of AT_1_R‐Ab (+) or (−) human sera samples or from control/standard samples of the CellTrend AT_1_R detection kit. (b) The affinity‐purified AT_1_R antigen was diluted to 1 μg/mL in PBS (pH 7.4) supplemented with protease inhibitor (Pierce Protease Inhibitor Mini Tablets, Cat# A32953) and coated overnight at 4°C with shaking on the Maleimide activated ELISA plates (Pierce Maleimide Activated Plates, Cat# 15150), blocked with ELISA blocking buffer for 1 h, and washed three times before testing.

### 2.5 ELISA Assay

Briefly, 100 μL of 100× diluted sera and AT_1_R‐specific primary antibodies were incubated on the prepared ELISA plates (described above) for 2 h at 4°C with gentle agitation. Any unbound or non‐AT1R‐specific antibody was removed by washing three times with PBS, and plates were incubated with secondary IgG‐conjugated HRP for 30 min, calorimetrically developed using Pierce TMB Substrate Kit (Cat# 34021) and measured using a Multiskan FC Microplate Photometer at 450 nm.

### 2.6 Antibody Adsorption With Crossmatch Cells and Elution (AXE) Procedure

The original method is explained in detail in the paper by Liwski et al. [26]. Briefly, Trex293 cells expressing AT_1_R were induced using 1 μg doxycycline in the presence of 5 mM sodium butyrate and harvested 48 h later. The number of cells was counted, and pellets were washed two times using PBS (pH 7.4). Cell pellets were incubated with ~200 μL of human serum positive for AT_1_R‐Ab (patient sera #1, 6, 7 and 8) for 30 min at 37°C with agitation. The supernatant was then retrieved and stored in −20, and the pellets were washed 6–7 times before antibody elution using the custom elution buffer (Glycine buffer pH 3.0, MgCl_2_) and neutralised using neutralisation buffer (Tris buffer pH 8.0). Following the buffer exchange with PBS (pH 7.4), the serum‐derived antibodies were subjected to incubation on either the CellTrend kit AT_1_R ELISA plate, including the kit's controls, or the in‐house prepared ELISA plate designed for the detection of anti AT_1_R‐Ab as previously described (in ELISA tray for detection of anti AT_1_R autoantibodies, part b).

### 2.7 Adsorb out Bead (AOB) Treatment

AOB ([OLI] Cat# ADSORB) consists of microparticles treated with blocking solution, but without any specific antigen coating, and is used to reduce the background reactivity due to nonspecific binding. The beads were used based on vendor‐provided protocol. Briefly, test serum and AOB were dispensed in a 10:1 ratio and incubated for 30 min at room temperature with agitation. The mixture was centrifuged for 5 min at 15,000 rpm, and the supernatant was transferred into a new tube without disturbing the pelleted beads. The sera are then transferred to the ELISA tray for further processing.

### 2.8 Effect of BSA Concentration on the CellTrend ELISA Signal Detection

BSA was diluted using either the kit's dilution buffer or PBS and added to the kit's standards and controls. The CellTrend standards refer to the 2.5, 5, 10, 20 and 40 U/mL. The positive control refers to a positive signal of ~20 U/mL, whereas the negative control should have a signal close to the blank. The BSA‐treated controls were incubated with the kit's ELISA tray and processed according to the vendor's protocol. In a separate experiment, the kit's microtiter plate was incubated with 0.01% or 0.001% BSA (in PBS) for 30 min at 4°C on a gentle shaker and washed once before the ELISA assay. To study the BSA inhibitory effect on signal detection on the CellTrend ELISA plate, various BSA dilutions ranging from 10^−2^% to 10^−6^% were added to the 100× diluted human sera and tested along with untreated sera and CellTrend standards.

## 3 Results

### 3.1 Detection Signal Interference With BSA and AOB

**FIGURE 1:**
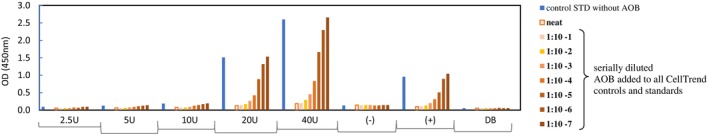
Effect of serial dilution of adsorb out bead (AOB)‐treated standards and controls on the CellTrend Kit Assay Tray (lot 64). AOBs used at different concentrations (10^−1^–10^−7^) in the CellTrend AT_1_R Kit's standards as well as positive (+) and negative (−) controls. At higher dilutions, the inhibitory effect is diminished. DB: CellTrend dilution buffer.

**FIGURE 2:**
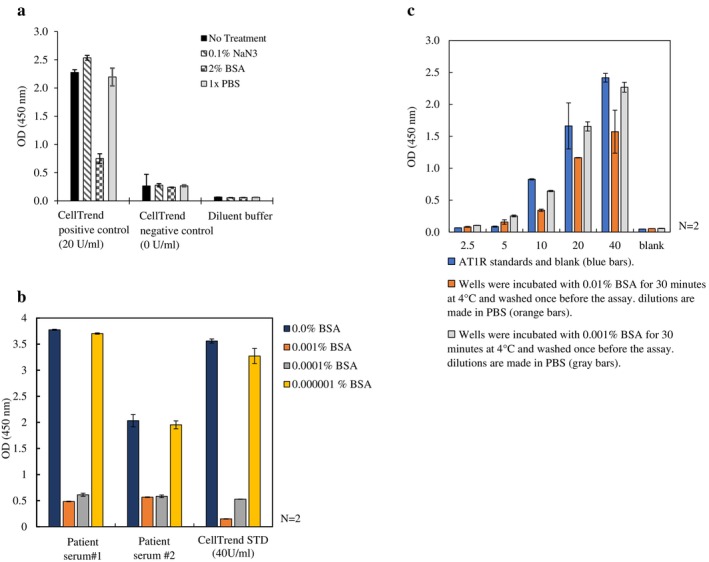
(a) BSA as the major component of AOB buffer impedes the detection signal in the positive control (~20 U/mL) of the CellTrend Kit, whereas other components do not interfere with the detection signal. (b) BSA titration effect on CellTrend positive control and AT1R Ab (+) human sera. Presence of BSA—even at very low concentrations—in the samples affects the signal detection on the CellTrend ELISA plate. Samples are incubated with various BSA concentrations before testing on the ELISA tray. (c) CellTrend AT1R kit standard detection signals restore after washing the BSA treated plate. The ELISA wells are washed after being exposed to the BSA (0.01% and 0.001% V/V in PBS). The signal is restored after washing the plate prior to the test. CellTrend standards contain 2.5, 5, 10, 20 and 40 U/mL, and the positive control corresponds to ~20 U/mL. *N* represents the number of replicas for each point.

Treating AT_1_R‐Ab (+) sera and CellTrend positive controls with AOB interfered with the detection signal, and the signals diminished with increasing AOB: sample titrations (Figure 1). Incubating the samples (human sera and CellTrend kit controls) with BSA (as little as 1 μg/mL) blocked the detection signals but had no effect when using 0.1% NaN_3_, PBS and kit dilution buffer (Figure 2a). The same effect was observed when AT_1_R‐Ab (+) human sera were incubated with various BSA concentrations (Figure 2b). Upon incubating the ELISA tray with various BSA concentrations for 30 min, blocked detection signals can be fully restored upon washing the ELISA tray and applying the CellTrend kit's controls (Figure 2c).

### 3.2 AT_1_R Expression and Purification

**FIGURE 3:**
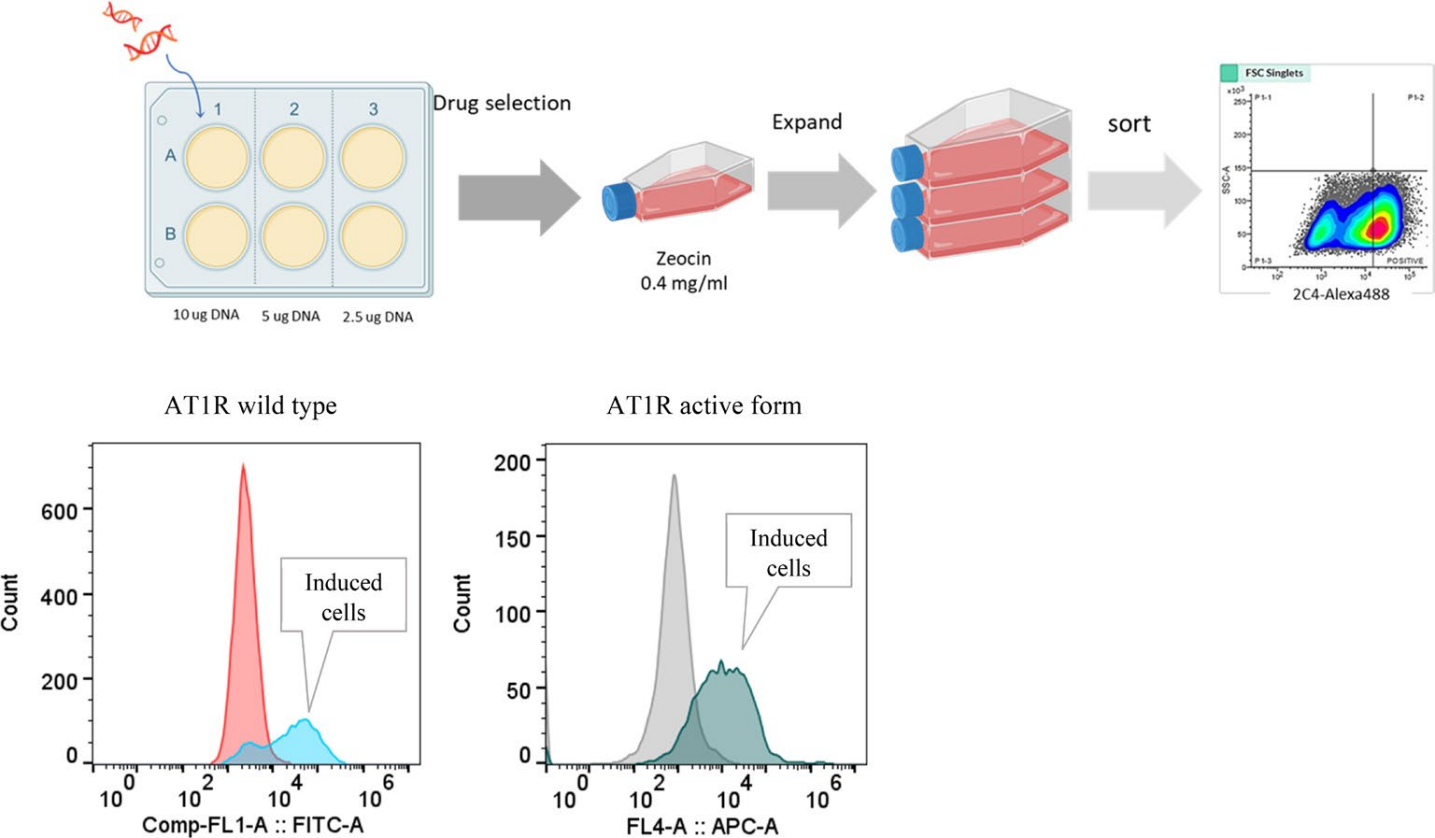
Establishing and verifying cells for AT_1_R expression: T‐Rex 293 cells were transfected with AT_1_R plasmid. Doxycycline‐induced cells are assayed for AT_1_R surface expression using either Alexa‐488 (wild‐type AT_1_R) or Alexa 647‐conjugated (active form) AT_1_R‐specific monoclonal antibodies.

Trex‐293 cells that were induced and analysed for AT_1_R expression exhibited a clear shift compared to control (uninduced) cells, as demonstrated in Figure 3, confirming the presence of AT_1_R on the cell surface. The purified liquid batch was sent for additional validation through MS–LC analysis (Supporting Information).

### 3.3 Verification of the Prepared ELISA Trays

**FIGURE 4:**
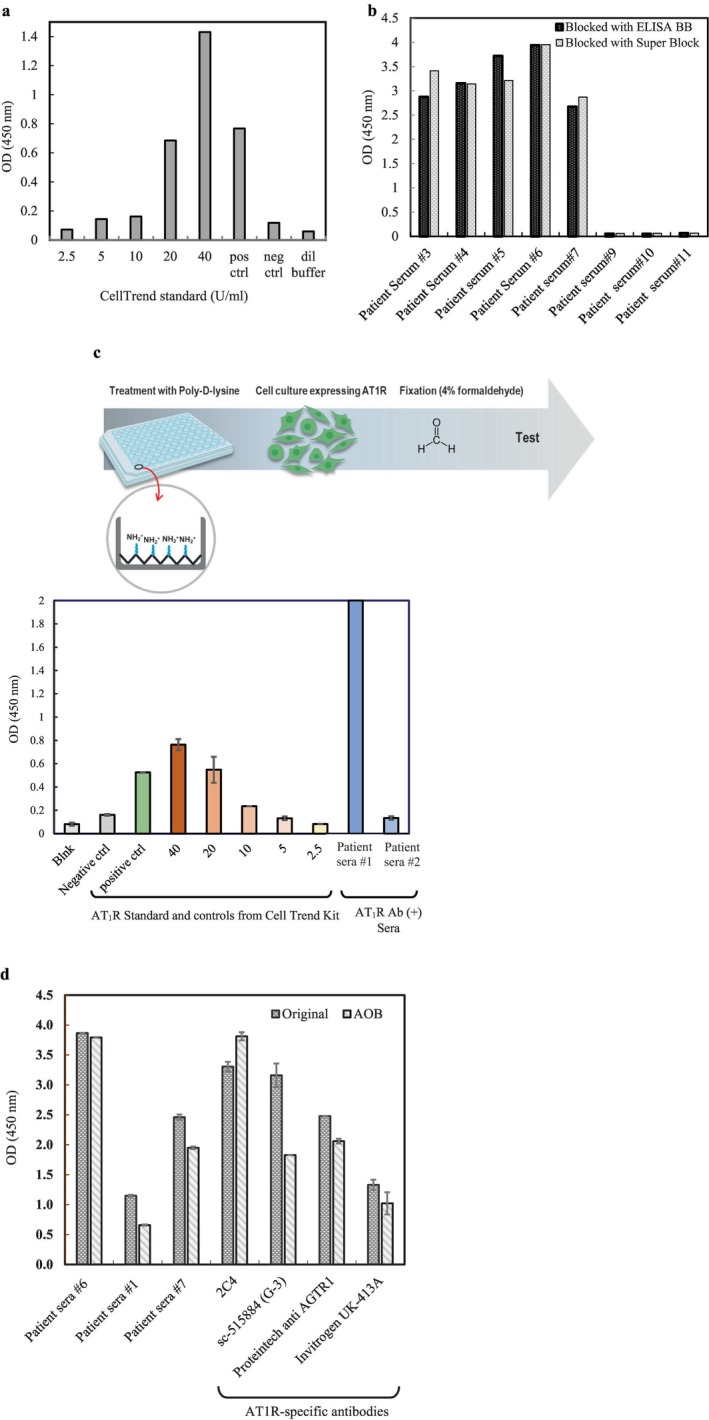
(a) CellTrend standards and controls tested on in‐house AT_1_R active form coated on ELISA plate. (b) Positive (#3–7) and negative (#9–11) human sera for AT_1_R antibody are tested on in‐house ELISA plate (AT_1_R active form). The in‐house AT_1_R active form is coated on a pre‐activated ELISA tray. Human sera, CellTrend standards (2.5, 5, 10, 20 and 40 U/mL), CellTrend positive (20 U/mL) and negative controls are tested on the AT1R‐coated ELISA tray. (c) T‐Rex293 cell expressing AT1R active form are coated and fixed on 96 well plate. Standards and controls from Cell Trend AT_1_R kit as well as patients' sera reacted to the ELISA tray coated with T‐Rex 293 Cells expressing AT_1_R active form, confirming the specificity of expressed AT_1_R antigen on the cell surface. (d) Internal AT1R ELISA plate. In‐house AT_1_R‐coated ELISA plates incubated with AOB treated samples. Signal inhibition was not observed with the samples treated with AOB (unlike CellTrend ELISA), indicating that AOB or BSA component are not posing an inhibitory effect on the in‐house AT_1_R coated ELISA tray.

To assess the functionality of the inducible cells and the affinity‐purified AT_1_R antigen‐coated ELISA trays in binding AT_1_R‐specific antibodies (AT_1_R‐Ab), ELISA plates coated with either AT_1_R expressing cells (Figure 4c) or affinity‐purified AT_1_R antigen (Figure 4a,b) were employed. The results revealed the ability of these ELISA plates to capture AT_1_R‐Abs from human sera or CellTrend kit control samples, shown in Figure 4a,b. When samples were treated with AOB according to the manufacturer's protocol (described in Section 2) and tested on the in‐house AT_1_R‐coated ELISA tray, no signal suppression was observed (Figure 4d), as opposed to CellTrend's ELISA tray (Figures 1 and 2a,b). The reason for this observation could be that the commercial kit uses the whole cell lysate for coating on the ELISA tray surface [27] and the other components present in the cell lysate may potentially cross‐react with the components in the sera or BSA.

### 3.4 Eluted AT_1_R‐Specific Antibodies From AT_1_R‐Ab (+) Serum React With the CellTrend ELISA Tray

**FIGURE 5:**
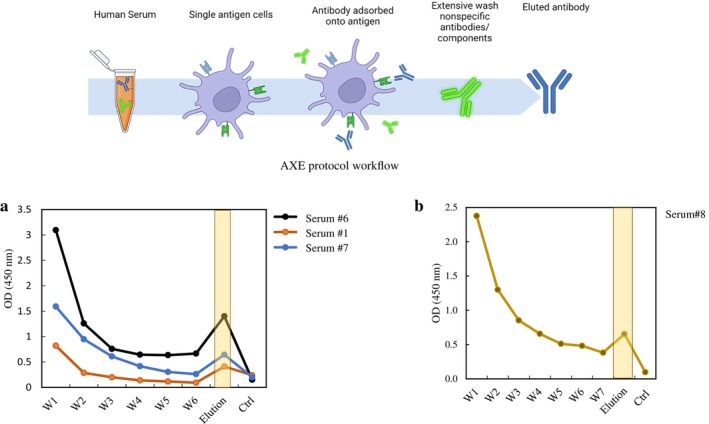
(a) AXE experiment: AT1R‐Ab (+) serum incubation and elution from AT1R active form tested on the CellTrend ELISA plate. W1–W6 correspond to the wash fractions. (b) AXE experiment: AT1R‐Ab (+) serum incubation and elution from AT1R active form tested on the in‐house AT1R ELISA plate (active form). W1–W7 correspond to wash fractions. Using AXE method [26], eluted antibodies from patient serum positive for AT_1_R were captured on AT_1_R active form expressing cells, and cross reacted to AT_1_R ELISA tray from the CellTrend kit and the in‐house active form AT_1_R coated ELISA plate.

The application of the AXE method [26] involved the isolation of AT_1_R‐specific antibodies from human sera that tested positive for AT1R‐Ab and had been absorbed by Trex‐293 cells expressing AT_1_R. The reactivity of these isolated antibodies was subsequently verified using either the ELISA tray provided by the CellTrend kit (Figure 5a) or the in‐house developed ELISA tray (Figure 5b). The results presented in Figure 5a,b demonstrate a notable reduction in absorbance at 450 nm (OD_450_) after successive washes of the cell pellet, particularly evident by Washes 6 and 7 when compared to the initial wash. Following the elution step, as described in Section 2, the absorbance of the eluted fraction containing the AT_1_R‐Ab is approximately 15%–50% of the OD_450_ in the standard Celltrend assay. This observation confirms our AT_1_R expression cell system and the specificity of the CellTrend AT_1_R ELISA platform. The same protocol was employed for the in‐house AT_1_R ELISA plate, confirming the reactivity of the eluted AT_1_R‐Ab with both platforms, despite the inherent dissimilarities between the two ELISA systems.

## 4 Discussion

This report aims to investigate the possible reasons behind the loss of AT_1_R‐Ab reactivity signal on the CellTrend ELISA (OLI), and to compare its performance with the newly developed (in‐house) affinity‐purified AT_1_R ELISA tray. Establishing and utilising the CellTrend AT_1_R‐Ab detection kit has been instrumental in assisting clinicians in identifying patients at high risk for transplant rejection, thereby improving clinical assessment outcomes in post‐transplant settings [1, 4, 8, 14, 15, 28, 29, 30, 31]. Although the current ELISA kit has not been cleared by the US Food and Drug Administration (FDA) for in vitro diagnostics use, the kit has been widely adopted in US‐certified diagnostics laboratories for over a decade.

A major challenge in serological diagnostic assays is the presence of background signals, which can contribute to false positives or false negatives. Therefore, reducing non‐specific binding is crucial for improving assay specificity. In anti‐HLA antibody detection, one approach to mitigating background signals is the use of adsorption with unconjugated latex beads (AOB), which effectively removes non‐specific interactions in Luminex‐based assays. However, AOB is not validated for use in ELISA platforms, and previous studies have reported that pre‐treating samples with AOB prior to ELISA assays may lead to a significant reduction or complete loss of detection signals (reviewed in Xu et al. [20]).

In the current study, we identified BSA—a component of the AOB buffer—as the primary factor responsible for signal interference in the CellTrend AT_1_R ELISA kit. This conclusion was supported by our observation that neither phosphate buffer nor 0.1% NaN_3_, other components of the AOB buffer, caused signal loss. Furthermore, we found that diluting BSA by a factor of 1 million neutralised this effect. Importantly, the BSA interference resulted from its interaction with serum components rather than the ELISA plate itself, as washing the plate after exposure to different BSA concentrations restored the detection signal. Unlike the CellTrend kit's ELISA using the whole CHO cell lysate [12, 14], our in‐house ELISA platform, which uses purified AT_1_R antigen, did not exhibit signal reduction following AOB treatment. This suggests that differences in the AT_1_R antigen presentation may contribute to the observed variations in assay performance.

To further assess the sensitivity and specificity of the two ELISA platforms, we performed experiments using the adsorption with crossmatch cells and elution (AXE) technique. This method selectively isolates antibodies bound to their target antigen on intact cells [26]. In our study, using a limited number of AT_1_R‐Ab positive human sera (confirmed for bioreactivity), we observed that cells expressing the active form of AT_1_R showed stronger binding to AT_1_R‐Abs than those expressing the wild‐type (inactive) AT_1_R. This was evident when the eluted antibodies were tested on both the CellTrend and in‐house ELISA platforms (Figure 5). While these findings suggest increased antibody reactivity toward the active AT_1_R conformation, the variability in elution efficiency (15%–50% based on OD_450_ readings) and the limited sample size warrant further validation in a larger cohort. Notably, in HLA antibody detection, the standard AXE assay typically achieves elution efficiencies between 30% and 60%, depending on the antigen's expression level. Our observed range (15%–50%) is consistent with prior reports, especially considering the limited sera available for testing.

In summary, our findings highlight that BSA in the AOB buffer interferes with AT_1_R‐Ab detection in the CellTrend kit's ELISA, primarily through interactions with serum components rather than with the ELISA plate surface. However, this interference was not observed in our in‐house developed ELISA, which utilises purified AT_1_R antigen. Additionally, our AXE experiments confirmed that both ELISA platforms effectively detect eluted antibodies specific to the active form of AT_1_R, supporting their sensitivity and specificity.

Despite these insights, there are important limitations to consider. First, our study was constrained by a limited number of AT_1_R‐Ab (+) human sera available for the AXE experiment. Second, validation was performed using CellTrend‐defined positive and negative controls, which, while useful, may not comprehensively capture all potential assay variations. Nonetheless, we ensured that positive control human sera (> 40 U/mL) derived from HLA DSA negative ABMR cases supports the relevance of our findings.

Given the observed effects of AOB buffer components and the necessity for adherence to established protocols, we emphasise that any modifications to the AT_1_R kit assay—such as the use of unapproved reagents—may lead to unintended and difficult‐to‐interpret results. Future studies should focus on expanding the sample size, refining antigen presentation, and further optimising assay conditions to ensure robust and reproducible AT_1_R antibody detection.

## Conflicts of Interest

Dr. Carroll has received travel bursaries to attend OLI annual HLA and Transplant workshops.

## Supporting information


**Data S1.** Supporting Information.

## Data Availability

The data that supports the findings of this study are available in Supporting Information of this article.
