# Supplementary material for: Interrogation of Interfering Factors in ELISA Detecting Angiotensin Receptor Antibodies and Specificity Validation Using the Adsorption Elution Crossmatch (AXE) Technique
Source: HLA. 2025 Jun 8;105(6):e70268. doi: 10.1111/tan.70268 (PMC12146235; doi:10.1111/tan.70268)
Supplement: Supplementary file 1 — Data S1. Supporting Information. [file TAN-105-e70268-s001.pdf]

Supplementary Material

Trex-293 cells expressing AT$_1$R-active mutant, 48 hours post induction. Used for AXE experiment and pellet collection.

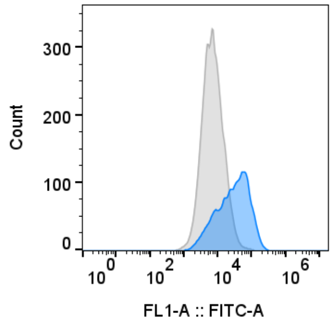

Sample Name	Count	Mean : FL1-A
A02 INDUCED F77A.fcs	4797	40928
A01 TREX293.fcs	8924	8792

Figure 5-supplementary data:
CellTrend AT$_1$R kit standards and controls on the CellTrend and in-house AT1R-coated Elisa plate (active form).

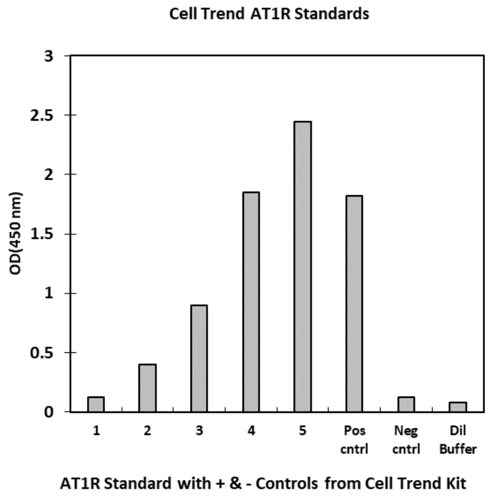

CellTrend kit standards on purified active form AT$_1$R coated plate

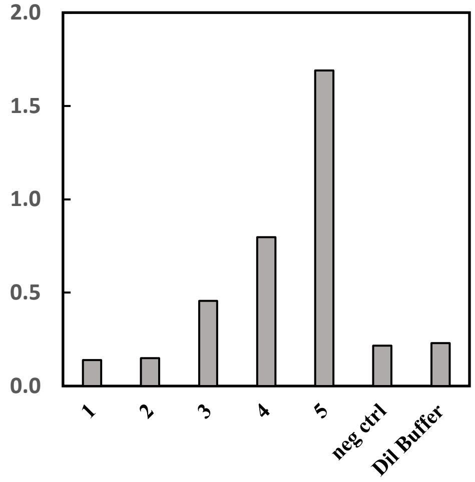

The elution fraction was sent as liquid samples for LC/MS:

KARAMAFROOZA_F77A_050823					
Hits	Protein Mass	No. of Peptide	Sequence Header	Link	Relative Abundance
1	42051.85	18	>sp|P60709|ACTB_HUMAN Actin, cytoplasmic 1 OS=Homo sapiens GN=ACTB PE=1 SV=1	P60709	26.2%
2	51702.35	10	>tr|U5LP42|U5LP42_MOUSE Anti anitbody H5M9 heavy chain (Fragment) OS=Mus musculus PE=1 SV=1	U5LP42	22.5%
3	41604.2	9	>sp|P30556|AGTR1_HUMAN Type OS=Homo sapiens GN=AGTR1 PE=1 SV=1	P30556	30.2%
4	54311.35	6	>sp|Q15233|NONO_HUMAN Non POU domain containing octamer-binding protein OS=Homo sapiens GN=NONO PE=1 SV=4	Q15233	3.1%
5	61187.5	5	>sp|P10809|CH60_HUMAN 60 kDa heat shock protein, mitochondrial OS=Homo sapiens GN=HSPD1 PE=1 SV=2	P10809	1.6%
6	67982.09	4	>sp|P27824|CALX_HUMAN Calnexin OS=Homo sapiens GN=CANX PE=1 SV=2	P27824	1.3%
7		4	>sp|P14866|HNRPL_HUMAN Heterogeneous nuclear	P14866	1.4%

AXE method
AT$_1$R-Ab (+) serum (patient serum#7) incubation and elution from Trex-293 cells expressing wild type AT$_1$R is tested on the CellTrend Elisa plate.
W1-W6 correspond to wash fractions.

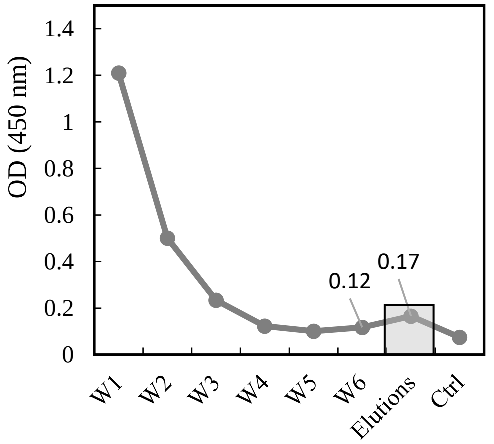

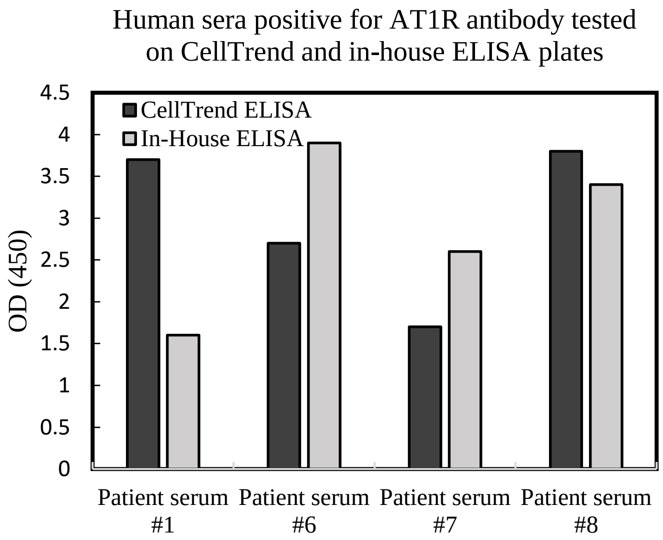

Table 1: Comparison of the detection signal ($OD_{450\ nm}$) of the patient sera positive for AT_1R-antibody between the CellTrend and in-house ELISA platforms:

ID in manuscript	CellTrend ELISA (OD 450)	In-house ELISA (OD 450)
Patient serum #1	3.7	1.6
Patient serum #2	2.3	-
Patient serum #3	1.7	2.8
Patient serum #4	2.1	3.1
Patient serum #5	3.9	3.7
Patient serum #6	2.7	3.9
Patient serum #7	1.7	2.6
Patient serum #8	3.8	3.4
